# Lectin spatial immunolocalization during *in vitro* capacitation in *Tursiops truncatus* spermatozoa

**DOI:** 10.21451/1984-3143-AR2019-0083

**Published:** 2020-02-05

**Authors:** Laura Robles-Gómez, María del Carmen Fuentes-Albero, Natalia Huerta-Retamal, Paula Sáez-Espinosa, Daniel García-Párraga, Alejandro Romero, María José Gómez-Torres

**Affiliations:** 1 Departamento de Biotecnología, Universidad de Alicante, Alicante, España; 2 Departamento de Biología, Avanqua-Oceanogràfic S.L., Valencia, España; 3 Departamento Investigación, Fundación Oceanogràfic, Valencia, España; 4 Cátedra Human Fertility, Universidad de Alicante, Alicante, España

**Keywords:** bottlenose dolphin, sperm capacitation, lectin

## Abstract

Spermatozoa interactions with the female reproductive tract and oocyte are regulated by surface molecules such as glycocalyx. The capacitation process comprises molecular and structural modifications which increase *zona pellucida* binding affinity. Lectins allowed us to describe glycocalyx changes during maturation, capacitation and acrosome reaction. This study had as its aim to identify lectin binding patterns using four lectins with different carbohydrate affinity in bottlenose dolphin (*Tursiops truncatus*) spermatozoa both before and after *in vitro* capacitation. Two semen samples from the same dolphin obtained on consecutive days were used, with four different lectin binding patterns becoming visible in both samples before and after capacitation. A highly stained equatorial segment with prolongations at the edges appeared as the most frequent pattern with *Wheat germ agglutinin* (WGA) in uncapacitated spermatozoa. However, it was homogeneously distributed over the acrosomal region after capacitation. Instead, the use of *Peanut agglutinin* (PNA) resulted in most spermatozoa showing high labelling in the acrosomal periphery region before capacitation and a homogeneous staining in the acrosomal region within the population of capacitated spermatozoa. Nevertheless, the most representative patterns with *Concavalin A* (ConA) and *Aleuria aurantia agglutinin* (AAA) lectins did not change before and after capacitation, labelling the acrosomal region periphery. These findings could contribute to the understanding of the reproductive biology of cetaceans and the improvement of sperm selection techniques.

## Introduction

Most of the interactions between the spermatozoon and its environment inevitably have their starting point in an interplay with the sperm glycoprotein and glycolipid covering; hence the tendency to associate the acquisition of a mature glycocalyx with the achievement of a full sperm fertilizing ability (Schröter et al., 1999). Sperm glycocalyx composition in mammals has already been studied by a variety of authors (Bearer and Friend, 1990; Schröter et al., 1999; Töpfer-Petersen, 1999; Tecle and Gagneux, 2015), most of whom used lectins. These molecules are proteins or glycoproteins of a non-immune nature that bind to specific membrane carbohydrate sequences which could be potentially involved in primary oocyte recognition (Osawa and Tsuji, 1987). The distribution of sugars in sperm glycocalyx has thus been described by means of various lectins, not only in different mammal species such as the goat (Bawa et al., 1993), the mouse (Baker et al., 2004), the rabbit (Nicolson et al., 1977), the monkey (Navaneetham et al., 1996) and the boar (Jiménez et al., 2002) but also, and especially, in humans (Lee and Damjanov, 1985; Gabriel et al., 1994; Fierro et al., 1996; Gómez-Torres et al., 2012). Likewise, studies suggest that the distribution of lectin receptors changes during spermatozoon epidydimal maturation, capacitation and acrosome reaction in mammals (Magargee et al., 1988; Bawa et al., 1993; Peláez and Long, 2007).

According to other authors, lectins may prove useful to select spermatozoon subpopulations with the highest fertilizing capacity (Gabriel et al., 1994; Purohit et al., 2008; Gómez-Torres et al., 2012), which stresses the usefulness of knowing how glycocalyx varies in other biologically or commercially valuable species. All this information could help increase the knowledge about the reproductive biology of endangered or vulnerable species, including cetaceans, which in turn can improve the set-up of artificial insemination (Robeck et al., 1998; Robeck et al., 2005).

*Tursiops truncatus* is currently listed in Appendix II, Annex A, of the Convention on International Trade in Endangered Species of Wild Fauna and Flora (Cites.org, 2019). In other words, although not enough data exist to categorize it as a species threatened with extinction worldwide, control needs to be exerted on its trade so that guaranteeing the survival of this species can remain the main priority. Even though breeding programs have been performed in this species thanks to the collaboration of aquatic parks, the difficulty to avoid consanguinity for the purpose of ensuring captive population sustainability has aroused great interest in the application of assisted reproduction techniques with the bottlenose dolphin (Andrews, 2000). For this reason, the knowledge of the molecular changes suffered by spermatozoa during *in vitro* capacitation could make it easier to select *T. truncatus* spermatozoon subpopulations with the highest fertilizing capacity for their use in artificial insemination processes. This would additionally facilitate genetic exchange along with the subsequent increase in genetic variability derived from maintaining group stability, avoiding the transport of animals or keeping a high number of breeding males in a single facility. Such measures can accordingly avoid further conflict and enhance the well-being of a dolphin group at reproduction time.

This study sought to describe the changes occurred in the glycocalyx of *T. truncatus* spermatozoa before and after *in vitro* capacitation using four lectins with different sugar affinity by fluorescence microscopy.

## Methods

### Semen collection

Ejaculated spermatozoon samples were obtained from a healthy adult bottlenose dolphin (*T. truncatus*) (which was 24 years old and weighed 187- kg) trained for voluntary semen collection (Keller, 1986) in Valencia’s Oceanographic. The collection was performed in accordance with the Animal Welfare Act for the care of Marine Mammals and in agreement with the Animal Care Protocol followed at the Oceanographic. Semen collection constitutes a routine medical behaviour —typically requested by trainers under veterinary supervision— for this species. Two samples obtained on consecutive days were used for this specific study after being transported to the laboratory keeping the temperature constant at 37ºC.

### Semen analysis

A basic semen analysis was performed in both samples —subsequently transferred to a sterile graduated container with the aim of determining the total volume and storage in an incubator at 37ºC for 30 minutes. As for pH, it was measured with a reactive strip.

A 20-micron Spermtrack chamber (Proiser R&D, Valencia, Spain) served to assess sperm concentration and a motility count was performed from *visu*. Mixing 5µl of semen with 5µl of the eosin solution on a tempered slide allowed us to evaluate sperm viability. The sample was left at rest for 30 seconds, monitoring the preparation in a phase contrast microscope. A total of 200 cells were evaluated in each sample and in each concentration, motility and viability analysis too.

### In-vitro capacitation by swim-up

**Figure 1:**
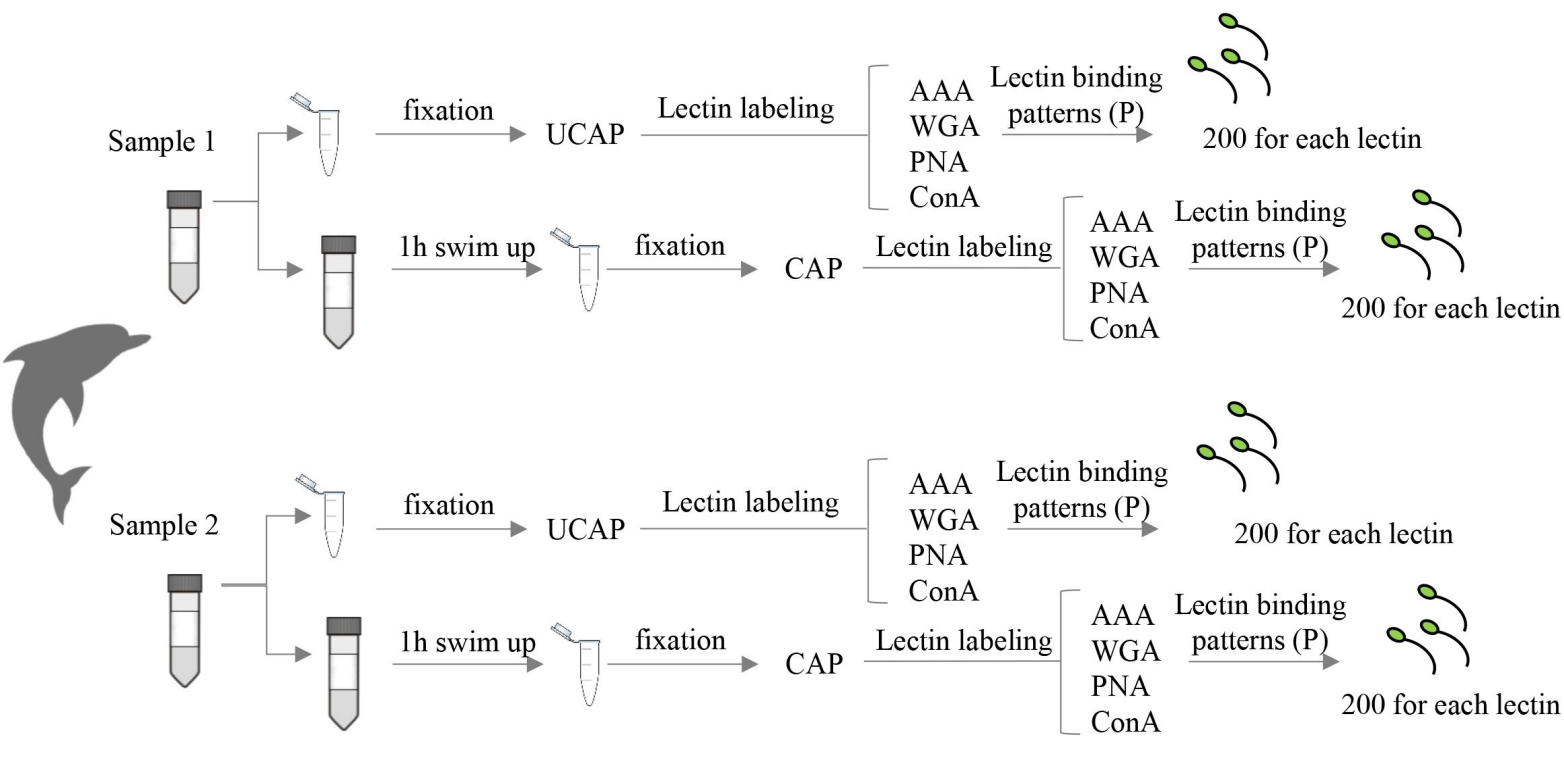
Experimental design used in this study.

At this point, sperm samples were divided into two physiological conditions: uncapacitated sperm (UCAP); and capacitated sperm (CAP) (Figure 1). Seminal plasma was removed after a ten-minute centrifugation at 250g and subsequently washed with Human Tubaric Fluid (HTF) (Origio, Malov, Denmark) for 5 minutes at 250g. Capacitation took place using the swim-up technique for 1 hour with a HTF medium supplemented by 5mg/mL bovine serum albumin (BSA) (Sigma, Madrid, Spain) at 37°C and 5% CO_2_.

### Sperm sample fixation

Samples before and after capacitation were fixed in a 2% paraformaldehyde (TAAB Essentials for Microscopy Ltd, Aldermaston, England) phosphate buffered saline solution (PBS) (Life Technologies, Grand Island NY, USA) for 1h at 4°C, after which they were diluted in PBS to a final concentration of 1 million cells/mL and stored at 4°C.

### Lectin labelling

Carbohydrate surface distribution in UCAP and CAP was characterized by means of AAA, ConA, PNA, and WGA lectins conjugated with FITC (Vector Laboratories, Burlingame CA, USA) (see Table 1 for the taxonomic name of sources and the specificity of lectins used). After being placed on a 10mm diameter round coverslip, fixed samples (5µL) were air-dried so that cells could attach to the surface. Afterwards, they were rehydrated with PBS for 10 minutes and incubated with a 2% BSA-PBS block solution for 30 minutes. An incubation of the coverslip with each FITC-conjugated lectin at a final concentration of 20µg/mL for 1h at room temperature in a humid chamber followed the blocking. Coverslips were later washed three times in PBS for 5 minutes each and assembled with Vectashield H-1000 with 4´6-diamidino-2-phenylindole (DAPI) (Vector laboratories, Burlingame CA, USA). Negative control experiments were performed omitting the lectin.

**Table 1:** Taxonomic names and specificity of lectins used in the present study.

**Name**	**Taxonomic name of source**	**Carbohydrate binding affinity**	**References**
AAA	*Aleuria aurantia*	Fuc-α(1.6)-GlcNAc	Osawa and Tsuji (1987)
		Fuc-α(1.2)-LacNAc	
ConA	*Canavalia ensiformis*	α-Man; α-Glc	Bhattacharyya et al. (1987)
PNA	*Arachis hypogaea*	Gal- β(1.3)-GalNAc	Lotan et al. (1975)
WGA	*Triticum vulgaris*	Neu5Ac; β-GlcNAc	Gallagher et al. (1985)

### Statistical analysis

We determined lectin binding patterns (P) through the assessment of 1,600 UCAP cells and 1,600 CAP cells (Figure 1) using fluorescence confocal microscopy (Leica TCS SP2 Microsystems GmBH, Wetzlar, Germany) and Leica Confocal Software. After examining every staining pattern identified with the different lectins, a decision was made to consider only those present in more than 5% of the spermatozoa in a sample, whether before or after capacitation. Statistical differences between UCAP and CAP spermatozoa were tested for the different lectins using a t-Student distribution. Differences were regarded as statistically significant at a 95% confidence level (P<0.05), the statistical analysis being performed with the 22.0 SPSS software version.

## Results

### Semen analysis

The seminal parameter values corresponding to both semen samples used in this study can be found in Table 2.

**Table 2:** Seminal parameters of the two semen samples of *Tursiops truncatus* used in this study.

**Seminal parameter**	**Sample 1**	**Sample 2**
Volume (mL)	20	45
pH	8	8.5
Concentration (million cells/mL)	450	250
Motility (%A+B)	90	60
Viability (%)	87	88

### Characterization of lectin labelling patterns in uncapacitated and capacitated sperm

**Figure 2:**
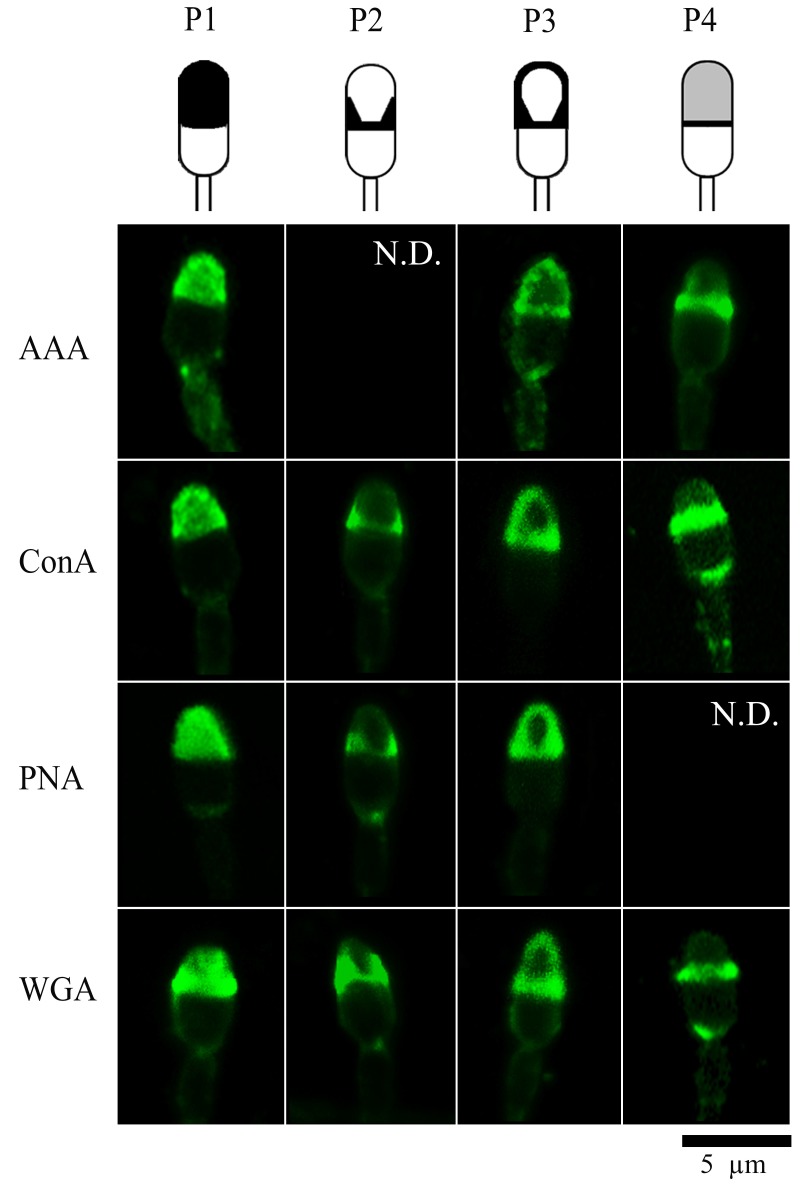
Observed lectins-binding patterns in bottlenose dolphin (*Tursiops truncatus*) sperm head in both experimental conditions (uncapacitated and capacitated). Confocal microscopy images at 63X magnification. Scale bar = 5µm. N.D: not detected. Pattern 1(P1): highly labelled acrosomal region; Pattern 2 (P2): equatorial segment stained with elevations at the edges; Pattern 3 (P3): edges of the acrosomal region highly labelled, and Pattern 4 (P4): highly stained equatorial segment and weak fluorescence in the acrosomal area.

Analyzing the lectin binding pattern in the head spermatozoa before and after capacitation allowed us to detect four seemingly consistent different patterns —shown in Figure 2. Lectin binding patterns were named as follows: Pattern 1(P1): highly labelled acrosomal region; Pattern 2 (P2): equatorial segment stained with elevations at the edges; Pattern 3 (P3): highly labelled edges of the acrosomal region, and Pattern 4 (P4): highly stained equatorial segment and weak fluorescence in the acrosomal region.

### Changes in lectin binding patterns after in vitro capacitation

**Figure 3:**
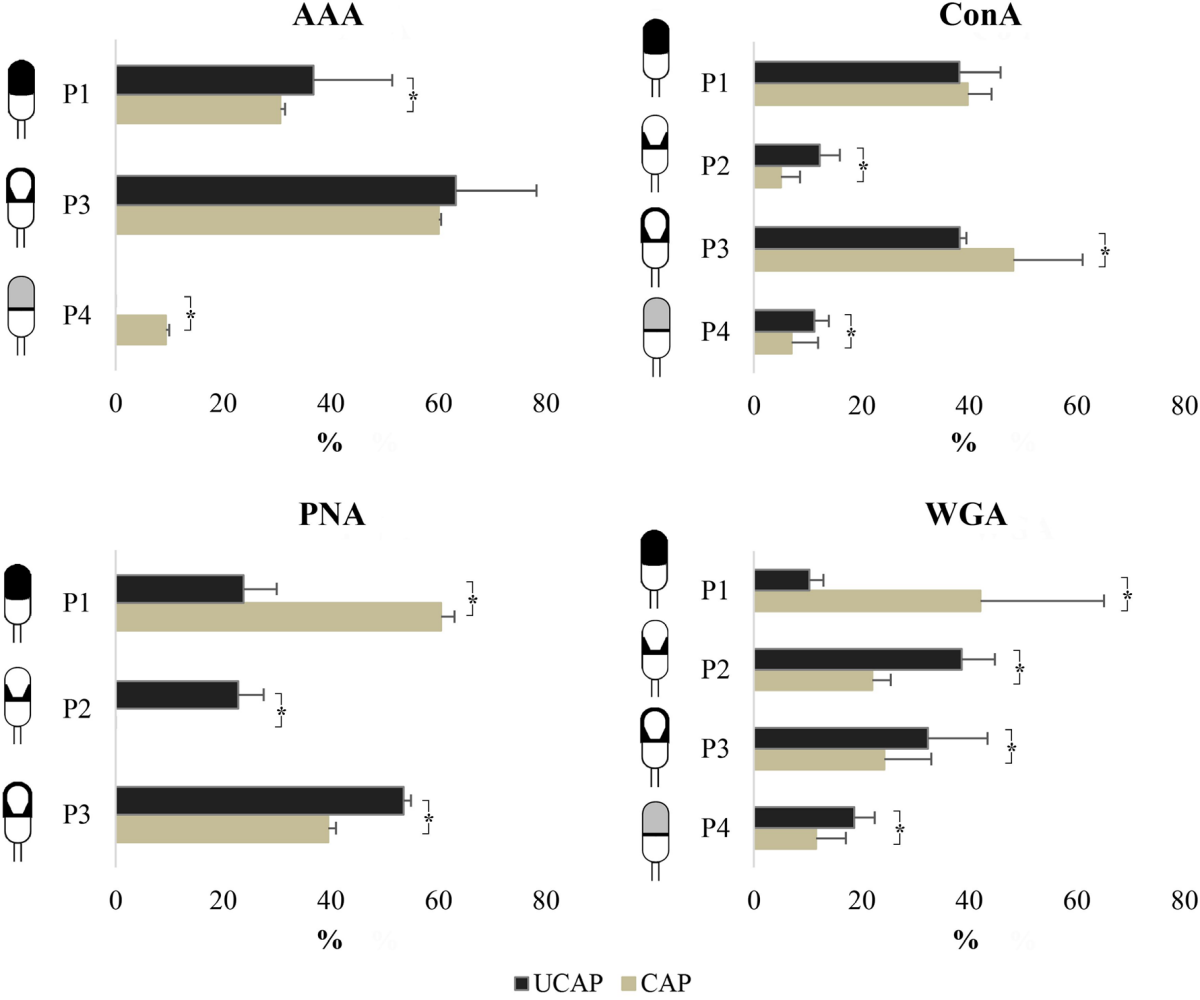
Differences in labelling patterns between uncapacitated sperm (UCAP) and* in vitro* capacitated sperm (CAP). Asterisk indicates significant differences between UCAP and CAP (P<0.05).

The most frequent pattern with AAA lectin both uncapacitated (63.25%) and in capacitated spermatozoa (60.06%) was P3. Significant differences were additionally identified in pattern P1 between cells before and after *in vitro* capacitation (36.75% and 30.63%, respectively). Regarding P4, despite not being observed in uncapacitated samples, it appeared at 9.31% of cells after the swim-up (Figure 3).

The same as with AAA lectin, the most abundant pattern after capacitation with ConA lectin turned out to be P3 (48.25%), this pattern being the most representative one in non-capacitated sperm (38.25%) too. Patterns P2 (12.25%) and P4 (11.25%) significantly changed following *in vitro* capacitation, though, lowering frequency to 5.00% and 7.00%, respectively. No statistically significant differences appeared in pattern P1 after capacitation (Figure 3).

Most of the uncapacitated sperm showed pattern P3 (53.50%), which largely diminished to 39.50% after *in vitro* capacitation, with PNA lectin. Instead, pattern P1 stood out as the most representative in capacitated cells (60.50%) —being significantly lower in uncapacitated spermatozoa (23.75%). P2 was present in 22.75% of cells prior to *in vitro* capacitation, but it disappeared after the swim-up process (Figure 3).

The percentage of WGA lectin patterns and their variation rate (Figure 3) showed that the P1 percentage increased to a great extent after capacitation —from 10.33% to 42.16%— this pattern being the most frequent one in capacitated spermatozoa. Whereas P2 appeared in 38.67% of cells before capacitation, its percentage significantly dropped to 22.01% in capacitated cells. As for the P3 percentage in uncapacitated and capacitated sperm, it reached 32.33% and 24.25% respectively, this difference being significant. Finally, P4 showed a sizeable decrease from 18.67% in non-capacitated sperm to 11.57% in capacitated sperm (Figure 3).

## Discussion

### Sperm subpopulations according to lectin patterns

Spermatozoon glycocalyx has been described by means of lectins in some mammal species (Nicolson et al., 1977; Bains et al., 1993; Bawa et al., 1993; Navaneetham et al., 1996; Jiménez et al., 2002; Baker et al., 2004). However, the composition and redistribution of spermatic glycocalyx during the capacitation process in *T. truncatus* still remains largely unknown. This report attests the presence of carbohydrates recognized by AAA, ConA, PNA and WGA lectins in bottlenose dolphin sperm glycocalyx before and after *in vitro* capacitation.

In relation to the total number of binding patterns identified (P1-P4), the results obtained in this work differ from those of other studies about sperm glycocalyx in other mammal species. By way of example, Gómez-Torres et al. (2012) identified seven patterns in human sperm using the same lectins. In turn, several studies have revealed that lectin receptor quantity and distribution varies between normozoospermic semen samples and oligozoospermic (Jiménez et al., 2002; Purohit et al. 2008) or teratozoospermic ones (Gabriel et al., 1994). In fact, according to Gabriel et al. (1994) a close relationship seems to exist between WGA receptors on human sperm membranes and sperm morphology. Therefore, we probably identified fewer patterns in *T. truncatus* sperm in contrast to human species due to the differences in the lower reference limit of sperm morphology between both species. The spermatic lower reference limit for normal sperm morphology in humans is 4% (WHO, 2010). Instead, normal morphology values of 90% have been described in previous studies dedicated to *T. truncatus* semen (Migliorisi et al., 2011; van der Horst et al., 2018), which suggests that seminal parameters could be better conserved in dolphins. The greater degree of homogeneity in bottlenose dolphin sperm morphology perhaps has to do with the fact that human sperm has more lectin binding patterns that bottlenose dolphin sperm.

### Immunolocalization of WGA and PNA

We observed four fluorescence binding patterns with WGA lectin (P1-P4). More specifically, patterns with highly stained acrosomal region and two prolongations towards the periphery of the acrosomal region (P2) stood out as the most frequent lectin distribution prior to capacitation. In contrast, receptors for WGA were homogeneously extended throughout the acrosomal region after capacitation (P1). Jiménez et al. (2002) unveiled a connection between WGA binding sites and fertility in boars, since WGA labelling was significantly lower in the spermatozoa of subfertile than in those of fertile boars (Jiménez et al., 2002). A similar redistribution appeared with PNA lectin. Uncapacitated spermatozoa showed a highly stained periphery of the acrosomal region (P3) but a homogeneous bound to the whole acrosomal region after capacitation (P1). This similarity between PNA and WGA patterns could derive from the arrangement of the glyceride residues with which they have affinity within glycocalyx. Regarding WGA lectin, which recognizes sialic acid and N-acetylglucosamine, it requires the presence of N-acetylneuraminic acid bound to galactose or N-acetylgalactosamine, carbohydrates with PNA affinity (Lassalle and Testart, 1994). Checking that WGA and PNA present similar and common patterns in our study should consequently come as no surprise. The distribution of the receptors is probably similar after *in vitro* capacitation because of the analogous distribution within glycocalyx. Furthermore, the molecular glycocalyx model proposed by Tecle and Gagneux (2015), shows that the sialic acid and N-acetylglucosamine are closely linked to galactose and N-acetylgalactosamine residues.

Added to this, the redistribution over the acrosomal region (P1) after *in vitro* capacitation observed with WGA and PNA lectin could correlate with a larger contact surface before oocyte recognition. Moreover, PNA lectin has been previously used to assess acrosomal status in *T. truncatus* (Montano et al., 2012). Therefore, the use of this lectin acts as a membrane integrity indicator and could be used for assessing the acrosomal morphology.

### Immunolocalization of AAA and ConA

In any case, our study did not reveal any significant differences between the most frequent patterns before and after capacitation with ConA and AAA lectins —which showed a highly stained periphery in the acrosomal region (P3). Perhaps mannose, glucose and fucose residues change in processes other than capacitation, such as gamete recognition, as exemplified by mice (Lee and Ahuja, 1987), or the methodology used prevents us from observing the changes occurred after *in vitro* capacitation. Fleming et al. (1981) argued that bottlenose dolphin spermatozoa were capable of fusing with zona-free hamster eggs only after preincubation for 2 hours, which leads us to think that the 1-hour-long incubation carried out in this study did not suffice to redistribute AAA and ConA receptors.

### Role of sperm glycocalyx during fertilization

It also deserves to be highlighted that fluorescence distribution in P3 might suggest what the morphology of the anterior region of the spermatozoon head in *T. truncatus* is like. Kita et al. (2001) described the anterior region of the sperm head in this bottlenose dolphin as being thin, flat and slightly concave using scanning electron microscopy (SEM). Moreover, field-emission scanning electron microscopy (FE-SEM) has provided higher resolution images of mammalian spermatozoa than conventional SEMs, thus permitting to observe cetacean spermatic morphology in detail (Meisner et al., 2005). Therefore, different spermatozoa membrane domains have been described in this clade —*e.g.* “apical ridge”, which refers to the marginal region of the anterior area of the sperm head. The peripheral zone which showed the P3 pattern in our study possibly corresponds to the elevated areas of the apical ridge.

In short, the specific distribution of lectin binding in bottlenose dolphin spermatozoa observed in this study definitely provides additional evidence not only about the presence of different domains in the plasma membrane surface but also about the changes that it experiences during *in vitro* capacitation.

Furthermore, two of the most common patterns identified in our results typically show a distribution at the periphery or at the boundary of membrane domains (P2 and P3). Studies on dog spermatozoa (Bains et al., 1993) and human spermatids (Lee and Damjanov, 1985) revealed a similar pattern, described as a “semilunar staining in the apical part of the acrosomal region”. An outstanding characteristic found in the male reproductive tract of dogs is the absence of seminal vesicles (Bains et al., 1993) —a peculiarity shared by dolphins (Harrison, 1969). These structures, along with the prostate, secrete glycoproteins which bind to the sperm surface in a selective way. Therefore, the fact that their glycocalyx has been formed without the influence of seminal vesicles probably explains the similar distribution of carbohydrates in dolphin spermatozoa and in human spermatids as well as in ejaculated dog spermatozoa.

Receptors in ejaculated bottlenose dolphin spermatozoa are generally found at the equatorial segment level, which makes sense if we remember that one of the main functions of glycocalyx is cell recognition until meeting the oocyte (Friend, 1982; Tecle and Gagneux, 2015). The binding patterns of each lectin can thus be linked to the head regions of greater interaction between the spermatozoon and its immediate environment. Even though many molecular aspects of the fertilization process still remain unknown when it comes to bottlenose dolphins, several authors have described the presence of longitudinal edges in the postacrosomal region (Fleming et al., 1981; Kita et al., 2001; Meisner et al., 2005) which could play a role in the fusion of the spermatozoon with the oocyte and / or in the early post-fusion (Fleming et al., 1981). Consequently, the initial recognition of the dolphin spermatozoa with the oocyte is likely to occur in the region of the equatorial segment, after which fusion takes place in the postacrosomal area.

Finally, since labelling with lectins could thus prove useful in selecting the sperm subpopulations with a fertilizing potential, the most common patterns of each physiological condition studied in this report could represent sperm subpopulations with a higher fertilizing capacity.

### Glycocalyx desing based on major AAA, ConA, PNA and WGA lectin patterns

**Figure 4:**
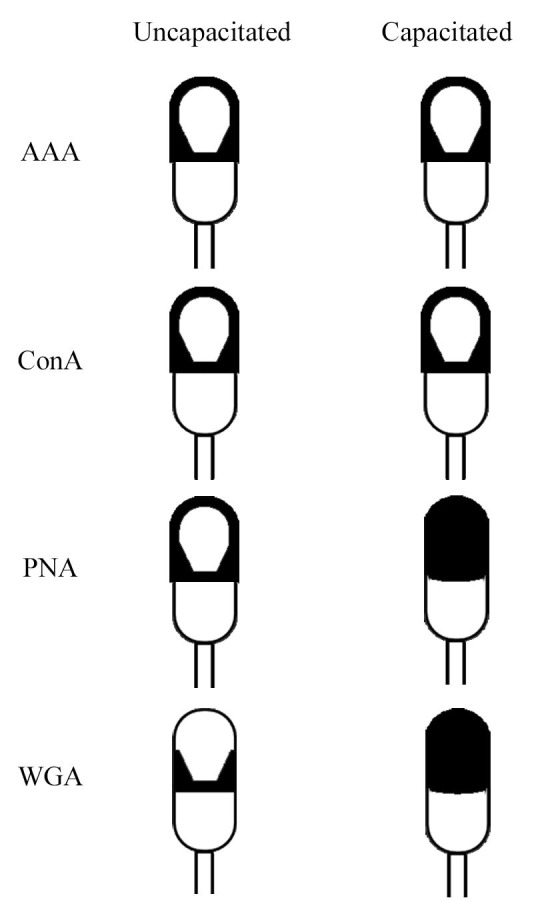
Glycocalyx sketch based on strong AAA, Con A, PNA and WGA lectin binding patterns observed in this study before and after *in vitro* capacitation in *Tursiops truncatus* spermatozoa.

In the light of all the above, we propose a model which represents the most representative location of different sugar surface in *T. truncatus* spermatozoa and its changes after *in vitro* capacitation (Figure 4). As shown by our model, the glycans recognized by means of AAA and ConA lectin do not change after capacitation and are distributed around the acrosomal region. However, those recognized by WGA and PNA appear in the periphery of the acrosomal region and in the equatorial segment in uncapacitated spermatozoa and throughout the acrosomal region after *in vitro* capacitation. According to these changes, WGA and PNA could be used as indicator of *in vitro* capacitation.

## Conclusion

These findings lead us to conclude that the labelling with WGA, PNA, ConA and AAA makes it possible to know the composition and distribution of sugars in the membrane of *T. truncatus* spermatozoa. Moreover, through the observation of the binding patterns in different stages; it could also evidence the presence of several sperm populations. According to this, it is possible the assessment of the fertilizing capacity based on the use of lectins. These findings would optimize artificial insemination in this species and improve the quality of life at the time of captive reproduction.
